# Poly(glycolide) multi-arm star polymers: Improved solubility via limited arm length

**DOI:** 10.3762/bjoc.6.67

**Published:** 2010-06-21

**Authors:** Florian K Wolf, Anna M Fischer, Holger Frey

**Affiliations:** 1 Institut für Organische Chemie, Johannes Gutenberg-Universität Mainz, Duesbergweg 10–14, D-55099 Mainz, Germany

**Keywords:** block copolymer, hyperbranched, PGA, polyester, polyglycerol, poly(glycolide), star polymer

## Abstract

Due to the low solubility of poly(glycolic acid) (PGA), its use is generally limited to the synthesis of random copolyesters with other hydroxy acids, such as lactic acid, or to applications that permit direct processing from the polymer melt. Insolubility is generally observed for PGA when the degree of polymerization exceeds 20. Here we present a strategy that allows the preparation of PGA-based multi-arm structures which significantly exceed the molecular weight of processable oligomeric linear PGA (<1000 g/mol). This was achieved by the use of a multifunctional hyperbranched polyglycerol (PG) macroinitiator and the tin(II)-2-ethylhexanoate catalyzed ring-opening polymerization of glycolide in the melt. With this strategy it is possible to combine high molecular weight with good molecular weight control (up to 16,000 g/mol, PDI = 1.4–1.7), resulting in PGA multi-arm star block copolymers containing more than 90 wt % GA. The successful linkage of PGA arms and PG core via this core first/grafting from strategy was confirmed by detailed NMR and SEC characterization. Various PG/glycolide ratios were employed to vary the length of the PGA arms. Besides fluorinated solvents, the materials were soluble in DMF and DMSO up to an average arm length of 12 glycolic acid units. Reduction in the *T*_g_ and the melting temperature compared to the homopolymer PGA should lead to simplified processing conditions. The findings contribute to broadening the range of biomedical applications of PGA.

## Introduction

Linear aliphatic polyesters such as polylactic acid (PLA) and poly(ε-caprolactone) [1] are of great interest due to their biodegradability, biocompatibility and permeability for many drugs. In contrast, poly(glycolic acid) (PGA) is scarcely used because of its high degree of crystallinity and its insolubility in all common solvents. However, glycolic acid is widely employed in copolymers of varying composition with the above-mentioned lactone comonomers [2]. For the PGA homopolymers, special processing techniques for the polymer melt are required and characterization is limited to solid-state techniques [3].

In recent works, PLA and poly(ε-caprolactone) [4] have been successfully used for the synthesis of numerous star [5] and multi-arm star [6] as well as (hyper)branched polymers [7]. Although PGA-rich polymers exhibit a superior degradation rate in comparison to poly(lactide), star copolymers primarily composed of this building unit have hardly been described in literature [8]. However, star copolymers, in a general sense, have attracted increasing interest for the fabrication of unimolecular micelles [9]; in particular when they consist of a hydrophilic, hyperbranched (or dendritic) core and a hydrophobic corona [10]. Their potential arises from their ability to encapsulate and release hydrophilic molecules slowly. Particularly, PEG/PLA-based copolymers have been intensely studied in this context [11–13]. Apart from this special application in solution, analogs of well-known linear polymers with star architectures exhibit significantly altered physical properties [14–15]. This is often considered the primary motivation for the choice of this interesting polymer architecture [16].

A suitable multifunctional core molecule is required to prepare multi-arm star polymers with core–shell characteristics. Apart from dendrimers [17–18], well-defined hyperbranched polymers [19] fulfill this requirement and benefit from their accessibility via a facile one-step synthesis, which makes a tedious, generation-wise build-up ubiquitous. Besides poly(ethylene imine) (PEI) [20], hyperbranched polyglycerol [21–26] has proven to be a versatile and highly potent multifunctional core molecule [27–29]. Derivatization and functionalization of the peripheral hydroxy groups of this polyether-polyol have afforded a number of carrier systems [30–34], matching the concept outlined above. In contrast to dendrimers, where functional groups are exclusively located at the surface, poly(glycerol) scaffolds also contain hydroxy groups throughout the structure. At first glance this might be considered a disadvantage, however, this is in fact beneficial for the significantly hydrophilized core environment when core–shell topologies for encapsulation are desired.

Here we present a solvent-free synthetic strategy for multi-arm star block copolymers with a hyperbranched polyether core and PGA arms, systematically varying arm length. The combination of glycolide with a multifunctional initiator studied in this paper is of a rather fundamental nature. Our primary objective is to improve the solubility of PGA in standard organic solvents and thus facilitate characterization as well as processing, while keeping the overall glycolide weight fraction high. Multi-arm star copolymers [35] should permit the combination of short average chain length with high molecular weight. Since the high number of functionalities of the core molecules is ideally translated into a matching number of arms with a respective chain end, end-group effects are expected to exert a significant influence on solubility and crystallization tendencies of the polymer.

## Results and Discussion

**Figure 1:**
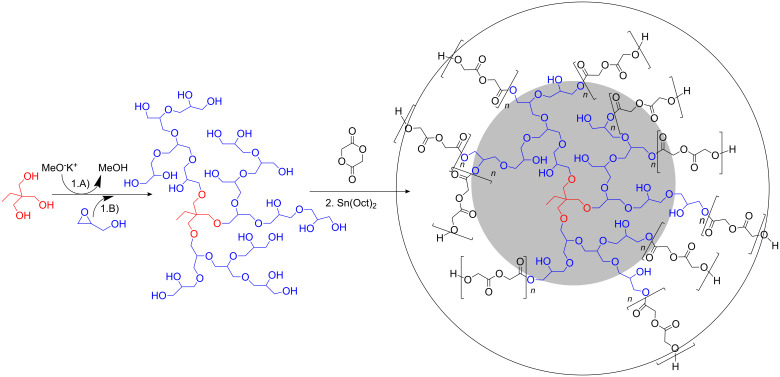
Synthetic route to *hb*-PG-*b*-(P)GA multi-arm star copolymers in a two-step sequence. The well-established anionic ring-opening multibranching polymerization of glycidol is followed by the Sn(Oct)_2_-mediated copolymerization of glycolide.

The hyperbranched poly(glycerol)s (PGs) with multiple poly(glycolide) arms were prepared by a straightforward two-step approach as shown in Figure 1. In the first step, we polymerized glycidol anionically by the method described previously [19], using trimethylolpropane as a trifunctional initiator. The hydroxy groups of PG were deprotonated to an extent of 10% before the slow addition of glycidol monomer was started. The subsequent polymerization proceeds via a ring-opening branching reaction where branching occurs due to a fast proton exchange equilibrium which is a well-known phenomenon in oxyanionic polymerizations.

In the second step, the polyether-polyols were used as macroinitiators for the ring-opening polymerization of glycolide via Sn(Oct)_2_ catalysis. All polymerization experiments were carried out in bulk (with a minimum of toluene present for transfer of the catalyst) at 120 °C for 24 h with systematic variation of the glycolide monomer/*hb*-PG-OH ratios. Since each glycidol unit leads to the formation of an additional end-group after ring opening and attachment to the growing PG, the corresponding total number of primary and secondary hydroxy groups of the polymer *n*(OH) is equal to the sum of the initiator functionality *f* and the degree of polymerization DP*_n_*.

[1]
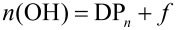


By varying of the initiator/monomer ratio, two hyperbranched poly(glycerol) samples with different degrees of polymerization DP*_n_* were obtained. Their theoretical number of initiating hydroxy groups was calculated from the degree of polymerization which is available from ^1^H NMR according to Equation 1. PG_14_ and PG_38_ thus offer an average of 17 and 41 potential initiating moieties for the grafting-from reaction with glycolide. It should be emphasized that according to Equation 1, the number of hydroxy groups is independent of the degree of branching (DB). Typically, the poly(glycerol) macroinitiators possess primary as well as secondary –OH groups, which likely show different reactivities in the initial reaction with glycolide. Since the accessibility of functional groups of PG is believed to play an important role in the properties of the resulting star block copolymer, the branched topology and the distribution of OH groups therein are key factors that will also be addressed in the following text.

Careful drying of the PG cores under vacuum is a crucial step for the controlled synthesis of the multi-arm star polymers in order to avoid initiation by trace amounts of water, which leads to concurrent glycolide homopolymerization and thus an undesired mixture of linear and star-like PGAs. In order to prevent possible precipitation from solution, the polymerization was conducted in bulk without added solvent under Sn(Oct)_2_ catalysis with an average catalyst loading of 0.1 mol % of the glycolide feed. The mixed compounds yielded a homogeneous melt at 120 °C, fulfilling a prerequisite for an efficient grafting-from approach. Under the reaction conditions employed and taking the high number of initiating groups into account, the conversion proceeds to high values within short reaction times. The polymers obtained show improved solubility properties compared to linear PGA and thus permit the use of common characterization methods such as NMR in DMSO-*d*_6_ and SEC in DMF. This is largely attributed to the high end-group concentration in combination with a short average PGA chain length in the multi-arm structure.

**Figure 2:**
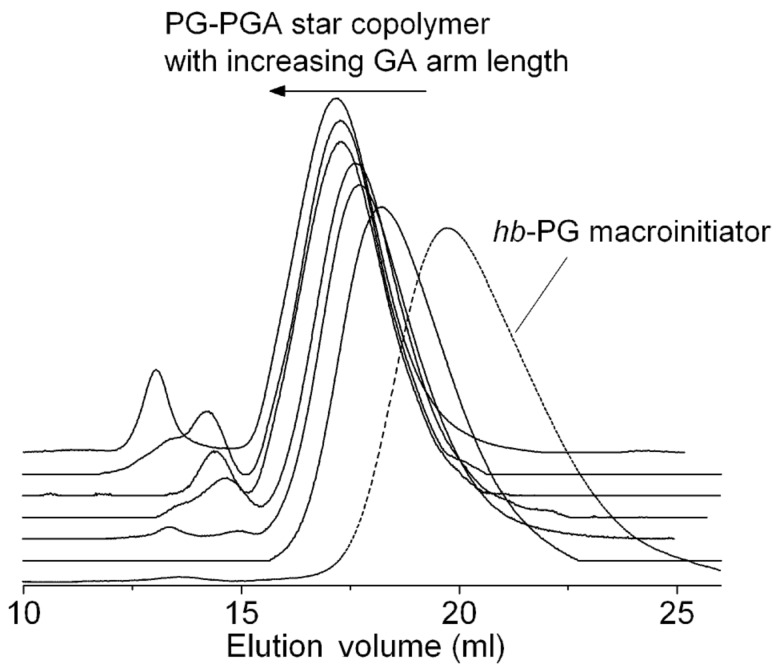
SEC-elugrams of the obtained multi-arm star block copolymers derived from PG_38_. The grafting of polyglycolide on the poly(glycerol) macroinitiator is accompanied by a significant decrease in elution volume.

With increasing glycolide content, a second high-molecular-weight distribution mode appears together with a gradual shift of the main distribution mode to lower elution volume (Figure 2) which is in line with expectations. These apparent impurities could be caused by compounds capable of co-initiation such as water or other hydroxy functionalities. Table 1 illustrates the correlation between theoretical molecular weight and values obtained from SEC measurements via calibration with polyethylene glycol (PEG) standards. The obvious underestimation of the molecular weight by SEC is attributed to the peculiar spherical geometry of the multi-arm star copolymer and has also been observed with other star polymers. The polydispersities of the materials are in the range of 1.3–1.7 for the series of star polymers prepared, which is moderate. These values can be explained by the non-monodisperse multifunctional initiator (PDI: 1.9–2.0), although transesterification/cyclization reactions during the synthesis cannot be completely excluded.

**Table 1:** Characterization data of the multi-arm star block copolymers originating from two different *hb*-PG macroinitiators from NMR and SEC.

sample	glycolide content (weight ratio)	yield (%)	*M*_n_ (theor./NMR*)	*M*_n_ (GPC)	PDI (GPC)	average arm length (NMR)

PG_14_	0	–	1,140*	1,130	2.0	–
P(G_14_GA_4_)	0.77	48	5,000	5,400	1.6	7
P(G_14_GA_8_)	0.87	90	8,800	6,500	1.5	10.6
P(G_14_GA_12_)	0.91	94	–	–	–	12.1
PG38	0	–	2,900*	2,450	1.9	–
P(G_38_GA_2_)	0.62	45	7,600	6,300	1.7	3.9
P(G_38_GA_4_)	0.76	72	12,300	9,300	1.5	5.6
P(G_38_GA_6_)	0.83	88	17,000	11,000	1.4	7.2
P(G_38_GA_8_)	0.87	83	21,700	14,300	1.5	8.6
P(G_38_GA_10_)	0.89	92	26,400	15,600	1.4	9.5
P(G_38_GA_12_)	0.91	93	31,100	17,000	1.3	9.8

A detailed account of the NMR studies aimed at determining the PGA arm length of the polymers is given in the following text. In this context, it should be emphasized that solubility in DMF and DMSO was generally limited to star polymers with targeted arm length of up to 12 glycolic acid units. Obviously, samples exceeding these values have not been characterized by SEC or NMR and are thus not listed in Table 1.

**Figure 3:**
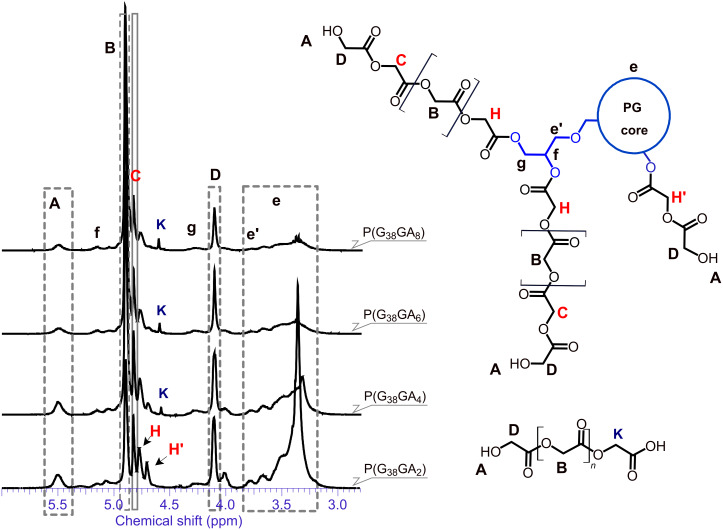
^1^H NMR analysis of the star block copolymers with increasing glycolide to poly(glycerol) ratio, using PG_38_ as core molecule.

**Figure 4:**
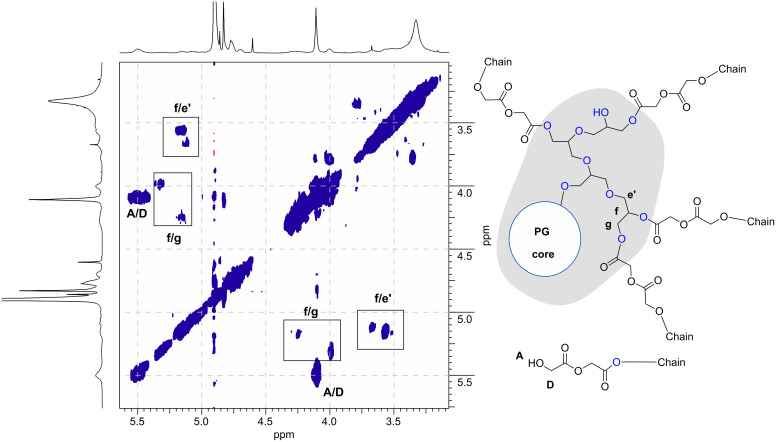
^1^H, ^1^H-correlation COSY NMR: this experiment visualizes correlations of terminal groups with their adjacent hydroxy groups as well as correlations within esterified glycerol units (**f**/**g** and **f**/**e′**). The most pronounced cross correlation peak can be assigned to the terminal hydroxy-methyl group of polyglycolide at 5.5/4.12 ppm (**A**/**D**).

The ^1^H NMR spectra of multi-arm polymer samples with varying composition (based on PG_38_) are shown in Figure 3. As expected, an increase in the glycolide feed results in an increase in the glycolide backbone signal at 4.91 ppm (**B**) and a relative decrease in signal intensity of the PG core. The resonances of the core are mainly distributed between 3.1 and 3.8 ppm (**e**). Special attention was paid to the terminal glycolic acid unit, since it enables the determination of the average chain length of the oligoglycolide arms. The respective signal can be found at 4.12 ppm and is thus well separated from the other glycolide arm-related signals **B**, **C**, **H** and **H′**. Furthermore, the signal denoted **A** at 5.5 ppm can be assigned to the terminal hydroxy group of the arms. This important signal assignment was confirmed by an ^1^H COSY NMR experiment (Figure 4), relying on the cross correlation of the methylene group **D** with the hydroxy proton **A**. Verification of the assignment of methylene and methine protons of the esterified primary and secondary OH groups of the PG core is crucial, since they evidence the successful linkage of arms and core. Unequivocal proof of attachment is obtained from the cross correlation of the methine/methylene proton of the major initiating species, the terminal glycerol units of *hb*-PG. Clear cross correlations between esterified secondary PG-OH (methine proton) groups (**f**) and esterified primary PG-OH (methylene proton) units (**g**) as well as esterified secondary PG-OH methine (**f**)/primary ether (**e′**) methylene protons can be observed. In the 2D NMR spectra of the star polymers, these protons have undergone a significant downfield shift (5.0–5.4 ppm), compared to the non-functionalized *hb*-PG-related signals, which are mainly found between 3.82 and 3.1 ppm. Although direct experimental proof could not be provided via 2D NMR, the signal denoted **C** at 4.84 ppm is assigned to the penultimate glycolic acid repeat unit. The first glycolic acid repeat unit, directly attached to the PG core, is represented by two different signals: **H** (4.78 ppm) or **H′** (4.72 ppm). While **H** corresponds to the first glycolic acid unit of a PGA chain, directly attached to the poly(glycerol) core, **H′** represents the special case of an α-unit of a glycolic acid dimer directly attached to the PG core (i.e. first and penultimate unit at the same time). Hence **H′** is predominantly observed for low glycolide fractions. This signal assignment is consistent with literature data for PGA-*co*-poly(ε-caprolactone) copolymers [36–37], as well as PLLA–PG star block copolymers which have recently been developed by our group [22].

The ^1^H COSY NMR spectrum further suggests that the linear and terminal glycolic acid units do not suffer from signal superposition and can thus be evaluated for the determination of the average arm length, which was achieved by the comparison of end-group- and backbone-related signals (**B** and **D**). Although a precise signal-to-structure correlation is difficult to establish, differentiation between PG and poly(glycolide) signals was achieved, confirming successful grafting of poly(glycolide) onto the PG core. Even more important, it was confirmed that the majority of the hydroxy groups of PG, particularly in the periphery of the core, was esterified.

An interesting correlation between the high-molecular-weight modes observed in SEC and the NMR spectra was found in the singlet, present at 4.61 ppm (**K**). According to literature data, this can be related to a carboxylic acid chain end of PGA homopolymer [38]. It can be observed for samples that exhibit an additional mode in SEC. This therefore supports the assumed formation of PGA homopolymer by co-initiation with water. Despite careful drying of the hygroscopic PG macroinitiator in vacuo, contamination with water could obviously not be fully eliminated. Since glycolide has been used as received and not stored in vacuo or under an inert gas atmosphere, this is the most likely cause for the introduction of traces of moisture into the system.

**Figure 5:**
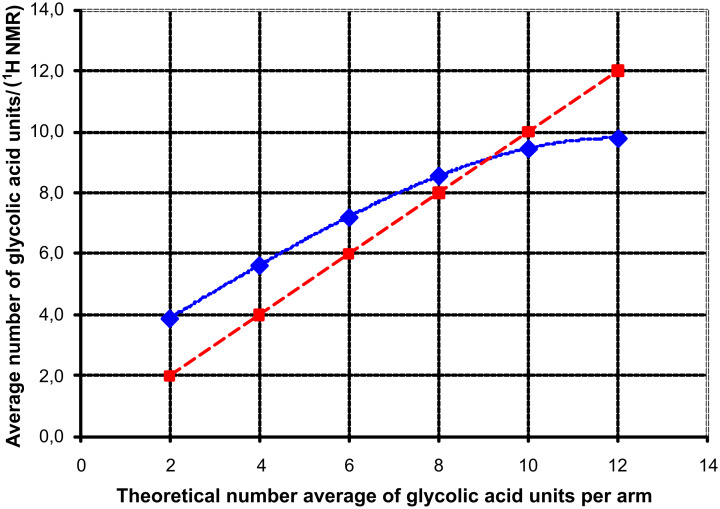
Comparison of average number of repeat units vs the theoretical number based on the ratio of PG and glycolic acid. The dotted line represents the ideal case of matching numbers and is added as a visual guide.

The graph shown in Figure 5 relates the number of glycolic acid repeat units per arm, calculated from ^1^H NMR for the series of PG_38_-derived star polymers. These values are compared with the theoretical number expected from the ratio of glycolide monomer to the sum of possible initiating sites in *hb*-PG.

Indeed, an interesting trend can be observed. This trend is most likely influenced by two factors:

1. For very low and moderate numbers of GA repeat units, the observed chain length of the glycolide stars exceeds theoretical expectations. This difference can be attributed to the difference in accessibility and nature (primary/secondary) of the hydroxy groups of the hyperbranched PG core. A certain fraction of potential initiating sites suffers from a reduced reactivity towards the employed glycolide lactone monomer. Especially, the hydroxy groups close to the core of the hyperbranched structure and/or those of a secondary nature are less active toward glycolide addition. The first ring-opening step of the glycolide lactone always generates/retains a primary hydroxy group which is more reactive for the attachment of further glycolide monomers than the average PG-hydroxy groups. Nevertheless, the observed yield of the precipitated star polymers (Table 1) was high enough to assume conversions exceeding 90% before the polymer melt congealed. In addition, ^1^H NMR spectra of the samples showed no residual glycolide monomer with its distinct singlet signal at 5.06 ppm (in DMSO-*d*_6_).
2. With increasing arm length, the observed number of units drops below the theoretical value. As stated above, we assume that water was introduced via the glycolide monomer (indicated by signal **K**). Hence, co-initiation by trace amounts of water increases with increase in the glycolide/macroinitiator ratio.

Since the effect discussed in the second postulate counteracts that in the first, we observe the described trend as an overestimation of the chain length rather than an underestimation.

**Figure 6:**
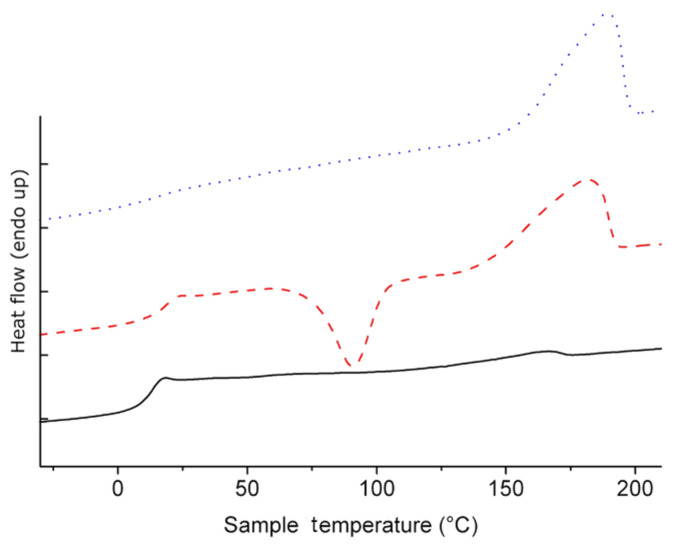
DSC heating traces (second heating run at 20 °C/min) for *hb*-PG_38_-*b*-GA_4_ (bottom), *hb*-PG_38_-*b*-GA_8_ (middle) and *hb*-PG_38_-*b*-GA_12_ (top), reflecting the increasing influence of polyglycolide chain length.

During the polymerization in the melt, continuous polymer melts with high viscosity are only observed for samples with a targeted average of up to 5–6 GA units. For longer arm lengths, the high mobility of the oligo-GA chains contributes to the consolidation of the melt via crystallization when reaching high conversion with a lack of molten glycolide monomer that can act as a plasticizer. This is supported by the results of the DSC measurements (Figure 6) of the star copolymers *hb*-PG_38_-*b*-GA_4_, *hb*-PG_38_-*b*-GA_8_ and *hb*-PG_38_-*b*-GA_12_ which confirm the variety of glycolide arm lengths achieved.

Generally, the observed glass transition (*T*_g_) of the glycolide arms for *hb*-PG_38_-*b*-GA_2_ and *hb*-PG_38_-*b*-GA_4_ is significantly depressed (10–18 °C) in comparison to literature data for PGA homopolymers [39] (approximately 40–50 °C). This reflects the influence of the flexible PG core. The *T*_g_ increased slightly with molecular weight, as it is also observed for most linear polymers. Both findings can be attributed to the low average number of repeating units per arm and are often observed for oligomers. This generally ensures increased chain mobility. As expected, this increased mobility enables efficient crystalline packing for a very short average chain length of 8.6 GA repeating units (for *hb*-PG_38_-*b*-GA_8_). Even for *hb*-PG_38_-*b*-GA_4_ with very low average PGA arm length a slight endothermic melting peak is visible in the DSC heating trace. The high crystallization tendency of the star block copolymers, despite the generally impeded crystallization due to the strongly branched PG core, is obvious from the data. An average chain length of less than 8–9 glycolic acid units is sufficient for a crystallization-induced vitrification of the polymer melt at 120 °C. The observed melting temperatures for star-shaped PGA range between 170 and 190 °C (Table 2) and are significantly depressed compared to PGA homopolymers of comparable molecular weight. This should allow polymer processing at lower temperatures which is, in particular, advantageous for such a thermolabile material.

**Table 2:** Thermal properties of selected multi-arm star copolymers.

samples	GA units/arm (theor.)	GA units/arm (found)	*T*_g_ (°C)	Δ*H*_m_ (J/g)	*T*_m_ (°C)

P(G_38_GA_4_)	4	5.6	10.13	3.1	161.4
P(G_38_GA_8_)	8	8.6	15.44	57.6	180.7
P(G_38_GA_12_)	12	9.8	17.5	59.9	189.5

## Conclusion

This work presents the first synthesis of star block copolymers based on glycerol and glycolide. *hb*-PG-*b*-PGA multi-arm star copolymers have been prepared via a core first approach, using hyperbranched poly(glycerol) with different hydroxy functionalities as core molecules. The melt copolymerization with *hb*-PG as macroinitiator via Sn(Oct)_2_ catalysis afforded well-defined complex polymer structures with predictable molecular weights. In contrast to their linear analogs of comparable molecular weight, the polymers exhibited superior solubility in organic solvents such as DMF and DMSO. This permitted detailed characterization via 1D and 2D NMR, SEC and DSC. It should be emphasized that the multi-arm star polymers presented possess molecular weights up to 31,000 g/mol and high glycolide weight content up to approximately 91 wt %. The short chain lengths of the oligoglycolic acid chains along with the increased number of end-groups are expected to enhance hydrolytic degradability significantly, rendering the novel materials promising candidates for drug release applications.

## Experimental

### Instrumentation

^1^H NMR spectra were recorded at 300 MHz on a Bruker AC 300. The spectra were measured in DMSO-*d*_6_ and the chemical shifts were referenced to residual solvent signals (^1^H proton NMR signal: 2.50 ppm). 2D NMR experiments were performed on a Bruker Avance-II-400 (400 MHz) equipped with an inverse multinuclear 5 mm probe head and a *z*-gradient coil. Standard pulse sequences for gs-COSY and gs-NOESY experiments were used. The refocusing delays for the inverse hetero-correlations were set to 3.45 and 62.5 ms, corresponding to ^1^*J*_C,H_ = 145 Hz and *^n^J*_C,H_ = 8 Hz, respectively.

For SEC measurements in DMF (containing 1 g/l of lithium bromide as an additive), an Agilent 1100 series was used as an integrated instrument, including a PSS Gral column (10^4^/10^4^/10^2^ Å porosity) and RI detector. Calibration was achieved with poly(ethylene glycol) standards provided by Polymer Standards Service (PSS)/Germany. Differential scanning calorimetry (DSC) measurements were carried out on a Perkin-Elmer 7 Series Thermal Analysis System with auto sampler in the temperature range from −40 to 230 °C at a heating rate of 20 K/min. The melting points of indium (*T*_m_ = 156.6 °C) and Millipore water (*T*_m_ = 0 °C) were used for calibration.

### Reagents

Diglyme (99%) and glycidol (Sigma Aldrich) were purified by vacuum distillation over CaH_2_ directly prior to use. Tetrahydrofuran (THF) was refluxed with sodium/benzophenone before distillation. Glycolide was purchased from Purac^®^/Gorinchem (Netherlands) and used as received. Tin(II)-2-ethylhexanoate (Sn(Oct)_2_), 97% was obtained from Acros and used as received.

The synthesis of *hb*-PG was conducted as described in previous publications, using the slow monomer addition technique [21,25–26].

**“Grafting from” polymerization of glycolide with hyperbranched polyglycerol-polyol as a macroinitiator.** In a typical experiment, exemplified for the synthesis of star block copolymers *hb*-PG_38_-*b*-GA_6_, 0.530 g *hb*-PG_38_ (0.181 mmol/ 7.33 mmol of primary and secondary hydroxy groups, according to Equation 1) was placed in a flask immersed in an oil bath at 120 °C and evacuated for at least 20 min. Glycolide 2.55 g (22.0 mmol) was charged into the flask, which was then re-immersed in the oil bath. To the homogeneous melt, 75 μL of a 10% solution of Sn(Oct)_2_ (0.022 mmol) was injected. The mixture was stirred vigorously under N_2_ atmosphere for 24 h. After cooling, the mixture was dissolved in hexafluoroisopropanol and precipitated in excess diethyl ether. The precipitation/purification process was carried out twice to yield pure polymer. The product was isolated by filtration and dried in vacuum at room temperature for 24 h to yield a white powder in all cases, except for the copolymer with an average targeted GA amount of two units per arm (P(G_38_GA_2_)), which was a viscous, non-transparent white oil.
